# Banana Powder as an Additive to Common Wheat Pasta

**DOI:** 10.3390/foods9010053

**Published:** 2020-01-05

**Authors:** Beata Biernacka, Dariusz Dziki, Renata Różyło, Urszula Gawlik-Dziki

**Affiliations:** 1 Department of Thermal Technology and Food Process Engineering, University of Life Sciences in Lublin, 31 Głęboka St., 20-612 Lublin, Poland; beata.biernacka@up.lublin.pl; 2 Department of Food Engineering and Machines, University of Life Sciences in Lublin, 28 Głęboka St., 20-612 Lublin, Poland; renata.rozylo@up.lublin.pl; 3 Department of Biochemistry and Food Chemistry, University of Life Sciences in Lublin, 8 Skromna St., 20-704 Lublin, Poland; urszula.gawlik@up.lublin.pl

**Keywords:** banana powder, drying, pasta, quality, texture, antioxidant capacity, colour

## Abstract

This study aimed to analyse the effect of dried banana powder (BP) on common wheat pasta characteristics. Wheat flour (type 500) was replaced with 1%, 2%, 3%, 4% and 5% of BP. Control pasta without BP addition was also prepared. Pasta quality parameters including texture, colour, cooking characteristics and sensory evaluation were determined. Total phenolics content and antioxidant activity were also evaluated. The increase in BP in the pasta recipe resulted in an increase in the weight increase index (from 2.88 to 3.55) and cooking loss (from 5.2% to 6.4%). The effects of the addition of bananas were also observed in changes in colour coordinates. It was shown that BP slightly decreased the lightness of cooked pasta and had little influence on colour coordinates of raw pasta. It was also found that the addition of BP higher than 3% decreased pasta firmness. The total phenolics content and antioxidant capacity of pasta increased with the addition of BP. Sensory evaluation of pasta showed that the replacement of common wheat flour with BP should not exceed 3%.

## 1. Introduction

Pasta is very popular in many countries because of its tastiness, nutritional characteristics and ease of preparation [1]. Because of its properties that accelerate metabolism, pasta is an excellent ingredient in a nutritional diet. This product is also valuable in some medical conditions such as type I and type II diabetes, as confirmed by clinical and scientific studies that have shown the superiority of pasta over other starchy products [2,3].

For the production of pasta, flours from various types of cereals are used. However, the best raw material is a small gruel obtained from durum wheat called semolina [4,5]. Compared with products obtained from common wheat flour, pasta made from semolina is characterized by a more yellow colour, resulting from a higher content of carotenoid pigments, higher resistance to overcooking and higher firmness [6]. Moreover, losses of dry substance during cooking are usually lower for products obtained from semolina [1,7]. However, for economic reasons, common wheat flour is often used for the production of pasta. Despite the poorer quality of products obtained from this raw material (less attractive colour and worse texture), it meets the appropriate quality requirements for flour and allows products with appropriate physical properties, and thus with acceptable culinary characteristics, to be obtained [6,8,9]. Fortifications are used to supplement some ingredients in pasta products and increase their nutritional value and quality. Often in pasta companies, supplements in the form of powdered dried vegetables or additives enriching the nutritional value are used to widen the range and increase attractiveness for consumers [1,9,10]. Recent studies have shown that a common wheat pasta can be enhanced with pro-health properties by the addition of bean [11], wheat bran [9], carob fibre (CF) [10], mushroom powder [1] and herbs [12]. Common wheat flour can also be enriched with ascorbic acid or citric acid. This allows undesirable colour changes to be counteracted and reduces the loss of natural dyes during the production and storage of common wheat pasta [13].

Banana (*Musa paradisiaca* L.), because of its good physiological properties, can be an interesting addition to pasta as it contains many minerals and numerous vitamins [14]. Bananas come from southeast Asia. There are approximately 80 species within Musa genus, among which banana-paradise or banana is the most common. It is a hybrid of probably two wild species, namely *Musa acuminata* and *Musa balbisiana* [15]. Individual varieties are used in small rural communities as special diets and in traditional medicine because of the health properties of these varieties [16]. It is one of the oldest plants grown in the tropical zone, where it is the main edible plant whose fruits often replace bread for people living in Africa, India or Indonesia [14]. Banana contains many important vitamins A, C, E, K and those from group Bas well as fibre and minerals such as magnesium, phosphorus, calcium and potassium [15,17,18]. The major part of the banana harvest is intended for consumption. The flesh is used for the production of flour, jelly, marmalade, jams, syrups and wines. The raw fruit pulp contains approximately 25% sugars, mainly sucrose and 7% starch, as well as essential oils, enzymes, pectins, vitamins B2, C, and E, niacin and provitamin A [16]. Although most bananas are consumed fresh, many products are made with the addition of unripe banana flour [19], green banana flour [18] and banana pulp [20]. Ovando-Martinez et al. [19] showed that pasta enriched with unripe banana flour is characterised by a low carbohydrate hydrolysis rate and may help to broaden the range of food products with a low glycaemic index.

The aim of the present study was to evaluate the effect of banana powder (BP) addition on quality and antioxidant properties of common wheat pasta.

## 2. Materials and Methods

### 2.1. Research Materials

Common wheat flour (WF) (type 500, protein 11%, fat 1.2%, carbohydrates 73%, fibre 3.2%, moisture content 11.8%, particles below 250 μm) obtained from Lubella (Lublin, Poland) was used for production of pasta. The ripe bananas used in the experiment came from Ecuador and were purchased from a local supermarket. BP had the following composition: 84.5% of carbohydrates, 9.0% of protein, 0.2% of lipid, 1.8 of fibre and about 4.5% moisture content. WF and BP were characterised following AOAC [21] methods for the content of carbohydrates, protein, lipid, fibre and moisture.

### 2.2. Banana Powder Preparation

Bananas were peeled and then cut into slices of approximately 5 mm thickness. The raw material was then frozen at −25 °C in a freezer (Liebherr GTL-4905, Bulle, Switzerland). Frozen samples (300 g, 76.6 moisture content (wb)) were lyophilised at 40 °C using an ALPHA 1–4 laboratory freeze-dryer with a drying chamber pressure of 63 Pa. During drying, the changes in mass sample were constantly recorded [22]. The dried raw banana (approximately 4% moisture content (wb)) was ground using a Retsh Grindomix GM 200 knife grinder. Particles of less than 0.2 mm have been accepted for flour [23]. The BP was stored in closed polyethylene bags in darkness at room temperature: 20–22 °C.

### 2.3. Pasta Preparation

The pasta-dough-making method is shown in Table 1. WF was replaced with BP at 1%, 2%, 3%, 4% and 5% (PB1, PB2, PB3, PB4 and PB5, respectively). The addition of an appropriate amount of BP was established on the basis of preliminary results. Control pasta (CP) was prepared without the addition of BP. The 500 g dough samples were formed and extruded using the KitchenAid Heavy Duty kitchen model 5KPM5. A hook mixer was used to prepare the pasta dough. All ingredients were mixed for 5 min (150 rpm). A matrix with round holes with a diameter of 2 mm was used.

The obtained pasta (vermicelli of approximately 3.0 mm thickness, 120 mm length) was layered on a pasta dryer (KitchenAid 5KPDR, Benton Harbor, United States y) and set in a climatic chamber (ICH 256, Düsseldorf, Germany) for 24 h at 25 °C and 20% relative humidity until the moisture of pasta reached between 11%–12% wb.

### 2.4. Physical Properties of the Pasta

#### 2.4.1. Colour

Colour coordinates (L*, a* and b*) of the flour mix with the addition of BP and of the pasta surface before and after cooking were measured using a CR-400C Chroma Meter (Minolta, Colour Lab, Osaka, Japan). TDC (total colour difference) was also calculated [24].

#### 2.4.2. Texture Analysis

A strength-tester machine—Zwick Roell BDO-FB0.5 TH (Zwick GmbH& Co., Ulm, Germany)—was used for pasta evaluation. The machine was equipped with a Warner-Bratzler’s knife (1 mm thick). The values of cutting forces for cooked pasta were recorded with appropriate software.

### 2.5. Hydration Properties

For each sample of raw pasta with different proportions of BP, WAI (water absorption index) and WSI (water solubility index) were determined. The analysis was carried out in accordance with Elkhalifa’s and Bernhardt’s methodology [25]. Powdered pasta samples (1.0 g) were added to 10 mL deionised water and mixed well. The samples were left to stand for 30 min, with intensive stirring, and the test tubes were then placed in centrifuge tubes. The samples were then centrifuged for 10 min with an acceleration of 3000 rpm. The supernatants were dried to constant weight at 105 °C. WAI and WSI were calculated according to Wójtowicz and Mościcki [26]:

(1) $$\mathrm{WAI}=\frac{\mathrm{Wet}\;\mathrm{sediment}\;\mathrm{weight}\;\left(g\right)}{\mathrm{Dry}\;\mathrm{sample}\;\mathrm{weight}\;\left(g\right)}$$

(2) $$\mathrm{WSI}=\frac{\mathrm{Dry}\;\mathrm{supernatant}\;\mathrm{weight}}{\mathrm{Dry}\;\mathrm{sample}\;\mathrm{weight}}\times 100\%$$

### 2.6. Total Phenolics Content and Antioxidant Properties

#### 2.6.1. Extract Preparation

To prepare the undigested buffer (phosphate-buffered saline) (BE) and water extract (WE), ground samples of dry pasta (1 g) were extracted according to the procedure described by Gawlik-Dziki et al. [27]. Phosphate-buffered saline (PBS) is a buffer solution commonly used in biological research. The osmolarity and ion concentrations of the solutions match those of the human body (isotonic). Thus, the use of the PBS buffer allows the extraction of potentially bioavailable compounds, while hydrophilic compounds are extracted using water.

#### 2.6.2. Total Phenolics Content

The total phenolics content (TPC) in cooked and uncooked pasta was determined according to Singleton and Rossi with slight modification [10]. TPC was expressed as GAE (gallic acid equivalent)/g d.m. (dry mass).

#### 2.6.3. Antioxidant Activity

Antiradical activity on 1,1-diphenyl-2-picrylhydrazylradical (DPPH) and radical scavenging activity against ABTS (2,2′-azino-bis(3-ethylbenzothiazoline-6-sulfonic acid)) were assessed using the method presented by Gawlik-Dziki et al. [27] and Re et al. [28], respectively, and expressed as EC50—extract concentration that provided 50% of activity [10]. The antioxidant activities were determined for both cooked and uncooked pasta samples.

### 2.7. Cooking Characteristics of Pasta

#### 2.7.1. Optimal Cooking Time (OCT)

Five hundred millilitres of tap water was poured into a vessel and boiled, and 50 g of pasta was added to the boiling water and mixed thoroughly. After 3 min, samples of boiling pasta were taken and crushed between transparent plates. This operation was repeated every 30 s, until the white core disappeared, which was evidence of incomplete cooking of the pasta. The time after which no white core was observed in crushed pasta is considered the minimum cooking time [9].

#### 2.7.2. Weight Increase Index (WI) and Cooking Loss (CL)

WI and CL were calculated according to the methods described by Bonomi et al. [29] and Biernacka et al. [10], respectively.

### 2.8. Sensory Evaluation of Raw and Cooked Pasta and Banana Powder

Sensory evaluation of raw pasta was carried out by assessing appearance and aroma. BP and pasta were evaluated using a 7-point hedonic scale. The consumer panel consisted of 54 members (30 males and 24 females, aged 20–47 years). The banana was evaluated in terms of its appearance, colour, aroma, taste and texture. Pasta for sensory evaluation was prepared for OCT. Cooked pasta was evaluated for aroma, colour, taste and consistency, expressed as firmness, adhesiveness and overall acceptability. When assessing the organoleptic characteristics, guidelines regarding cleanliness, adequate lighting and protection of the room from foreign smells were followed [30].

### 2.9. Statistical Analysis

One-way analysis of variance (ANOVA) and Tukey’s post-hoc test (α = 0.05) were used to compare groups (STATISTICA 6, StatSoft, Inc., Tulsa, USA). Three individual experiments for each kind of pasta prepared were performed.

## 3. Results and Discussion

### 3.1. Drying Curve for Banana

**Figure 1:**
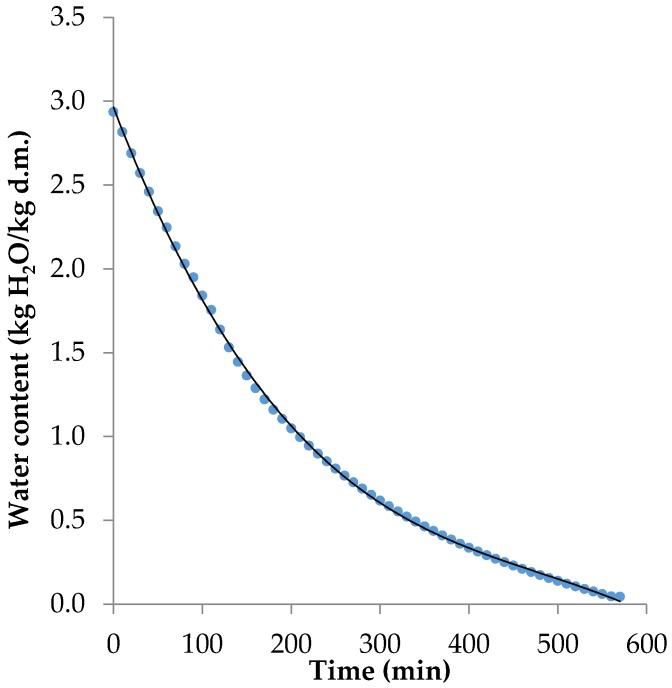
Freeze-drying curve of banana, d.m. (dry mass).

The freeze-drying curve for banana is presented in Figure 1. The time of drying the bananas at 40 °C from fresh state to approximately 4% moisture content was about 570 min. The highest drying rate was observed during approximately the first 150 min of dehydration. Afterwards, the drying process slowed down.

### 3.2. Colour Parameters

The colour parameters of raw and cooked pasta are presented in Table 2. Regarding coordinates of the colour of raw pasta, it was observed that BP had little influence on the lightness (L*) of the pasta. The minimum value for L* (64.32) was obtained for PB3 pasta, whereas the maximum value of this parameter (73.09) was found for the sample with 5% addition of banana. The colour component a* (redness) changed across a rather narrow range from 2.13 (control sample) to 2.60 (sample with 3% addition). Redness of CP was significantly lower from the redness of PB1, PB3 and PB5. The values for yellowness (b*) ranged from 10.55 (CP and PB3) to 12.30 (PB1). However, yellowness of CP was only significantly lower than b* of PB1 and PB2. Durum wheat pasta in comparison with common wheat pasta is usually characterised by lower values for lightness and redness but higher values for yellowness [31]. TDC was in a range from 3.53 to 4.79 for samples PB2 and PB4, respectively. It is assumed that a TDC value above 3.5 is recognisable [32], that is, each level of BP addition caused visible changes in raw pasta colour.

The lightness of cooked pasta decreased with the increase in the proportion of dried banana. The control sample was characterised by lightness at 71.97, while the lightness of samples with a 5% addition averaged 62.82. However, the addition of BP up to 3% had no significant influence on L* (Table 2). In a very recent study [20], banana flour was prepared from whole green banana and added to tagliatelle pasta at up to 30% substitution for wheat flour. Contrary to previous findings, there was no evidence of darker colour in banana pasta, which was possibly due to the use of wheat flour instead of semolina flour. The redness decreased after cooking the CP, and the same trend was observed for BP-enriched pasta. Addition of BP up to 2% had no significant influence on a*, whereas for PB3, PB4 and PB5, significantly higher values for redness were obtained. The yellowness colour component was characterised by different values between 9.10 (pasta with 5% of BP) and 12.18 (CP). Generally, PB addition caused a decrease in b* values. However, significant changes were observed only between CP and PB2, PB3, PB4 and PB5. The reduction in the intensity of the yellow colour of the cooked pasta may be due to the swelling of the pasta and the conversion of pigments, thereby causing a decrease in yellowness during cooking [33]. For cooked pasta, TDC was significantly increased with the addition of BP (from 1.44 to 9.70). The difference in colour between CP and pasta with BP was especially recognisable in PB4 and PB5.

### 3.3. Cutting Force

**Figure 2:**
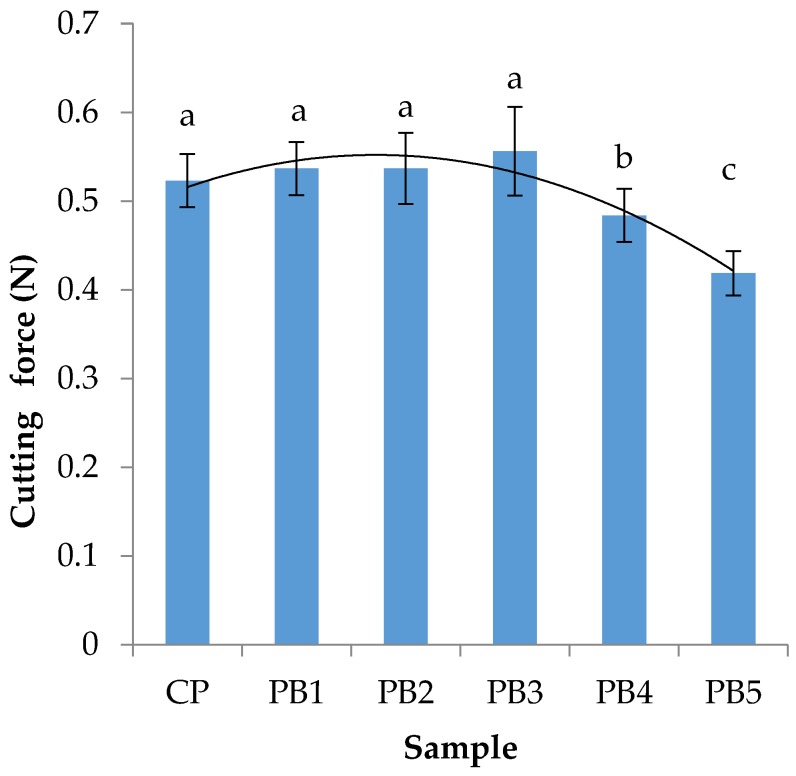
Relationship between cutting force of pasta and the amount of dried banana. PB1, PB2, PB3, PB4, PB5, pasta with 1%, 2%, 3%, 4% and 5% of dried and powered banana. Values followed by different letters are significantly different (*p* < 0.05).

The results for pasta cutting force (F_c_) are presented in Figure 2. F_c_ of pasta indirectly expresses its hardness. This parameter is one of the basic characteristics of the texture of this type of product [34].

F_c_ of cooked pasta ranged from 0.42 N to 0.56 N. It was observed that the addition of dried banana up to 3% did not have a significant influence on F_c_. Above this value, F_c_ decreased. A similar tendency was observed by Lisiecka et al. [33] when *Cistus incanus* L. leaves were added to common wheat pasta. According to previous research into different cereal products [20], the addition of banana flour did not affect textural properties and added nutritional value to food products. Dziki and Laskowski [35] showed that durum wheat pasta is characterised by higher F_c_ values in comparison with common wheat pasta.

### 3.4. Hydration Properties

Results for hydration properties of the pasta sample showed no significant difference in the water absorption index between pasta with BP and the control (Table 3). WAI is an important parameter of food as this food imbibes water without dissolving protein, thereby resulting in viscous and thick food products [3]. The WAI values were within the range of 7.27% to 7.43% (CP and pasta with 4% addition, respectively) and were not significantly different. Similar results for WAI were obtained for corn semolina supplemented with field bean semolina-enriched gluten-free pasta in the studies by Dib et al. [3]. Moreover, a reduction in WSI was observed compared with that of the control sample without BP. The increase in the addition of BP from 1% to 4% to pasta resulted in a slight but significant reduction of WSI from 43.94% to 40.06% (sample control and PB4). However, the differences in WSI for CP and PB5 were not significant.

### 3.5. TPC and Antioxidant Properties of Pasta

Table 4 presents the amounts of TPC in extracts of raw and cooked pasta. The lowest amount of TPC in raw pasta (average 9.4 and 16.0 mg GEA/g d.m., water and buffered extracts, respectively) was found in CP and PB1. Generally, as BP increased, TPC also increased. The highest polyphenols at concentrations of 13.4 and 30.3 mg GEA/g d.m. were found in pasta enriched with 5% BP addition (water and buffered extracts, respectively). A similar tendency has been found by other authors [33] in the study of pasta enriched with *Cistus incanus*. Studies of durum spaghetti with banana flour conducted by Ovando-Martinez et al. [19] also showed that TPC values obtained for pasta enriched with unripe banana flour were significantly higher than those obtained for CP, especially with respect to the content of condensed tannins. Cooking caused a significant decrease in TPC from 15% to 22%. A similar tendency was found for both water and buffered extracts. TPC in cooked pasta samples ranged from 7.1 to 10.4 mg GEA/g d.m. and from 12.0 to 25.9 mg GEA/g dm for water and buffered extracts, respectively. Other authors have also found a decrease in TPC after cooking of durum wheat pasta. Hirawan et al. [36] found a 39% overall decrease in TPC of spaghetti after cooking. However, the cooking time for the spaghetti was about 12 min. In our study, average cooking time was considerably shorter (average 5.5 min) and it is probable that this was the reason for the lower cooking loss.

The antioxidant activities of raw pasta determined by ABTS radical scavenging assays and expressed as ability to scavenge free DPPH radicals (EC50) values were from 41.3 to 33.0 mg dm/mL (pasta without banana and pasta with 5% addition in water extracts, respectively), and similar values were observed for the buffered extracts (EC50 ranged from 37.7 to 32.7 mg dm/mL for pasta without banana and pasta with 5% addition, respectively). Many authors have found that the enrichment of pasta with plant materials influences the increase in antiradical activity [12,14,33]. A similar trend was found in the results of the DPPH assay. The ability to scavenge free DPPH radicals (EC50) ranged from 150 to 332 mg dm/mL (pasta with 5% banana and control pasta in water extracts, respectively) and from 152 to 436 mg dm/mL (pasta with 5% banana and control pasta in buffered extracts, respectively). The highest antioxidant activity, for both extractants used, was found in sample PB5. Ovando-Martinez et al. [19] found that the addition of unripe banana flour to durum wheat pasta enhances antioxidant capacity.

Cooking caused a decrease in the antioxidant activity of pasta. As a result of this, the EC50 values increased. BP increased the antioxidant capacity of cooked pasta samples in both WE and BE extracts. In the case of ABTS, the EC50 values decreased from 46.8 to 37.2 mg dm/mL and from 42.3 to 36.1 mg dm/mL for WE and BE extracts, respectively. A similar tendency was observed for DPPH (EC50 changed from 353.4 to 223.3 mg dm/mL and from 458.0 to 212.8 mg dm/mL for WE and BE extracts, respectively). Based on the data in Table 4, it can be concluded that more compounds reacting with the Folin–Ciocalteau reagent were extracted using the PBS buffer. In the case of pasta, which is subjected to hydrothermal treatment prior to consumption, it seemed reasonable to use both water (to compare the content of hydrophilic compounds) and PBS (to determine the content of potentially bioavailable compounds). Other authors have also found a decrease in the antioxidant activity of durum wheat pasta as a result of cooking [36].

### 3.6. Cooking Properties

The average optimum cooking time (OCT) of pasta determined in the studies was ±5.5 min. The addition of BP to the wheat flour up to 4% had no significant influence on OCT, whereas for PB4 and PB5 samples a significant decrease in OCT was observed. An increase in BP in the pasta recipe increased the cooking loss and water absorption of pasta. In general, pasta with the addition of dried banana was characterised by higher WI and higher CL (Table 5). The values for these parameters ranged from 2.88 to 3.55 and from 5.2% to 6.4%, respectively. For good-quality pasta, cooking loss should not exceed 8% [19]. Other authors have also found that enrichment of pasta with other plant materials causes an increase in WI and CL [10,33]. Moreover, Sobota and Zarzycki [37] showed a similar level of CL in pasta produced from durum wheat semolina and common wheat flour.

### 3.7. Sensory Evaluation of BP

Dried bananas are characterized by a pleasant and specific aroma. The components responsible for BP odour are 3-methylbutyl ester, 3-methylbutyl acetate, 3-methylbutyl butane acid ester and 3-methylbutanoic acid [38]. BP received high scores for all sensory attributes, especially for aroma and external appearance (6.7 and 6.5, respectively) (Table 6).

### 3.8. Sensory Evaluation of Cooked Pasta

For the sensory evaluation of cooked pasta, the best scores in terms of colour were obtained for CP and PB1 pasta (Table 7). The increase in the addition of banana negatively affected the colour of pasta. The samples of pasta without the addition of BP and PB1 were judged to have the best colour (average 6.69), while pasta with 5% BP addition had the worst colour (3.16). In terms of smell, the evaluation team considered CP pasta and pasta with 1%, 2% and 3% addition of banana (average 6.1) to be the best, while pasta with 4% and 5% addition of banana was rated worse (4.16 and 4.80, respectively). The maximum value for taste was given to CP, PB2 and PB3 (average 6.19), and the minimum value was given to pasta with 5% BP addition (4.57). CP and pasta with up to 3% addition of banana showed the best firmness (average 6.32), and the worst firmness was obtained for pasta with 4% and 5% addition of dried and powdered banana (average 5.62). The increase in the addition of dried banana up to 4% had a slight or no influence on pasta adhesiveness. A higher addition of BP resulted in a decrease in adhesiveness. The worst score for firmness was received by pasta with 5% BP addition (5.67). In general, the addition of banana in pasta up to 3% favoured a positive evaluation for the majority of the traits studied and for the overall acceptability. A higher addition of BP did not improve the quality of enriched pasta. Generally, supplementation of a food product with health ingredients should not affect its palatability and consumer preferences. Along with the additional health benefits it offers, the product should be very good in terms of taste, smell and appearance [18]. According to Cheok et al. [39], pasta prepared with the addition of green banana varieties is recommended for consumers who prefer light pasta with a strong colour and a firm texture. In our study, we showed that BP from ripe bananas can also be used as a functional additive for common wheat pasta.

## 4. Conclusions

Enrichment of wheat flour with BP influences both the cooking parameters of pasta and its quality. The addition of dried banana at 4% and 5% resulted in a shortening of OCT and decrease in Fc. The best scores for overall sensory evaluation of pasta are obtained when the addition of BP does not exceed 3%. The addition of BP affects the total colour difference between raw and cooked pasta. In particular, an increase in the share of BP higher than 3% led to cooked pastas that were darker and slightly more red. Importantly, enrichment of common wheat pasta with BP resulted in an increase in TPC and antioxidant activity of the supplemented pasta. Moreover, the results showed that the replacement of wheat flour with dried banana should not exceed 3% in the production of common wheat pasta if acceptable sensory qualities are to be retained. Taking into account these results, the replacement of wheat flour with BP should not exceed 3%.

## Figures and Tables

**Table 1:** Pasta-making method.

Ingredients and Pasta-Making Conditions	CP	PB1	PB2	PB3	PB4	PB5
Wheat flour (g)	500	495	490	485	480	475
Banana powder (g)	0	5	10	15	20	25
Water	Up to the dough moisture content 45% (wb)					
Dough mixing time/speed	5 min/150 rpm					
Dough drying conditions	25 °C, RH 20%, 24 h					

CP—Control pasta, PB1, PB2, PB3, PB4, PB5, pasta with 1%, 2%, 3%, 4% and 5% of dried and powered banana, respectively, moisture content (wb).

**Table 2:** Colour parameters of raw and cooked pasta.

Sample	Raw/Cooked	Parameter			
		L*	a*	b*	TDC
CP	Raw	68.92 ± 0.81 ^a,b^	2.13 ± 0.15 ^a^	10.55 ± 0.45 ^a^	-
PB1		72.34 ± 0.44 ^b^	2.45 ± 0.03 ^b^	12.30 ± 0.59 ^b^	3.86 ± 0.25 ^a,b^
PB2		72.12 ± 1.71 ^b^	2.37 ± 0.11 ^a,b^	12.00 ± 0.39 ^b^	3.53 ± 0.48 ^a^
PB3		64.32 ± 3.60 ^a^	2.60 ± 0.08 ^b^	10.09 ± 0.34 ^a^	4.64 ± 0.52 ^b^
PB4		69.67 ± 3.06 ^a,b^	2.38 ± 0.07 ^a,b^	10.55 ± 0.41 ^a^	4.79 ± 0.43 ^b^
PB5		73.09 ± 0.76 ^b^	2.50 ± 0.15 ^b^	11.27 ± 0.70 ^a,b^	4.25 ± 0.37 ^b^
CP	Cooked	71.97 ± 0.55 ^c^	0.78 ± 0.06 ^a^	12.18 ± 0.27 ^b^	-
PB1		72.48 ± 1.53 ^c^	0.92 ± 0.14 ^a,b^	10.84 ± 0.53 ^a,b^	1.44 ± 0.57 ^a^
PB2		70.32 ± 0.50 ^b,c^	0.90 ± 0.02 ^a,b^	9.54 ± 1.13 ^a^	3.12 ± 0.31 ^b^
PB3		69.77 ± 1.64 ^b,c^	1.56 ± 0.25 ^c^	10.16 ± 0.90 ^a^	3.09 ± 0.55 ^b^
PB4		67.98 ± 0.85 ^b^	1.32 ± 0.12 ^b,c^	9.38 ± 0.89 ^a^	4.91 ± 0.46 ^c^
PB5		62.82 ± 1.50 ^a^	1.71 ± 0.17 ^c^	9.10 ± 0.16 ^a^	9.70 ± 0.64 ^d^

L*, a*, b*—Lightness, redness, and yellowness, respectively, TDC—Total colour difference, CP—Control pasta, PB1, PB2, PB3, PB4, PB5, pasta with 1%, 2%, 3%, 4% and 5% of dried and powered banana, respectively. Mean ± SD, *n* = 6, separate analysis of raw and cooked samples was performed, values followed by different letters are significantly different (*p* < 0.05).

**Table 3:** Results of hydration properties of the raw pasta sample.

Parameter/Sample	CP	PB1	PB2	PB3	PB4	PB5
WAI (g/g)	7.27 ± 0.19 ^a^	7.35 ± 0.02 ^a^	7.32 ± 0.03 ^a^	7.35 ± 0.01 ^a^	7.43 ± 0.06 ^a^	7.34 ± 0.01 ^a^
WSI (%)	43.94 ± 0.13 ^b^	40.62 ± 0.04 ^a^	41.53 ± 1.01 ^a^	41.54 ± 0.98 ^a^	41.06 ± 0.35 ^a^	42.13 ± 0.54 ^b^

WAI—Water absorption index (g/g), WSI—Water solubility index (%), CP—Control pasta with coarse-grained flour, PB1, PB2, PB3, PB4, PB5, pasta with 1%, 2%, 3%, 4% and 5% of dried and powered banana, respectively. Mean ± SD, *n* = 3. Values followed by the same letter in the same row are not significantly different (*p* < 0.05).

**Table 4:** TPC (total phenolics content) and antioxidant activities of raw and cooked pasta.

Sample	Total Phenolics Content and Antioxidant Activity	Water Extract (WE)		Buffered Extract (BE)	
		Raw	Cooked	Raw	Cooked
CP	Total phenolics content (mg GEA/g d.m.)	9.2 ± 0.43 ^B,a^	7.1 ± 0.27 ^A,a,b^	15.4 ± 0.67 ^D,a^	12.0 ± 0.15 ^C,a^
PB1		9.6 ± 0.45 ^B,a^	7.5 ± 0.19 ^A,a,b^	16.6 ± 0.58 ^D,a^	13.3 ± 0.32 ^C,b^
PB2		9.9 ± 0.51 ^B,b^	7.9 ± 0.31 ^A,b^	19.5 ± 0.75 ^D,b^	16.5 ± 0.20 ^C,c^
PB3		10.1 ± 0.64 ^B,b^	8.5 ± 0.19 ^A,c^	24.3 ± 0.94 ^D,c^	19.6 ± 0.57 ^C,d^
PB4		11.3 ± 0.72 ^B,c^	9.6 ± 0.17 ^A,d^	26.6 ± 1.20^D,d^	21.7 ± 0.63 ^C,e^
PB5		13.4 ± 0.85 ^B,d^	10.4 ± 0.24 ^A,e^	30.3 ± 2.30 ^D,e^	25.9 ± 0.79 ^C,f^
CP	Radical scavenging activity ABTS EC50 (mg d.m./mL)	41.3 ± 2.16 ^B,d^	46.8 ± 2.21 ^C,e^	37.7 ± 1.15 ^A,d^	42.3 ± 2.21 ^B,e^
PB1		40.2 ± 1.95 ^B,c,d^	45.6 ± 1.56 ^C,e^	36.8 ± 1.63 ^A,d^	41.5 ± 1.05 ^B,d,e^
PB2		38.9 ± 1.52 ^B,c^	42.1 ± 0.89 ^C,d^	35.7 ± 0.94 ^A,c,d^	40.4 ± 0.52 ^B,C,d^
PB3		36.5 ± 1.63 ^A,b^	40.0 ± 0.73 ^B,c^	34.3 ± 1.15 ^A,a,b,c^	39.3 ± 0.27 ^B,c^
PB4		34.9 ± 1.27 ^A,a,b^	37.6 ± 1.02 ^B,b^	33.9 ± 0.98 ^A,a,b^	38.7 ± 0.66 ^B,b^
PB5		33.0 ± 1.41 ^A,a^	37.2 ± 0.86 ^B,a^	32.7 ± 0.86 ^A,a^	36.1 ± 0.42 ^B,a^
CP	Antiradical activity, DPPH EC50 (mg d.m./mL)	332.4 ± 15.2 ^A,e^	353.4 ± 13.4 ^B,e^	436.0 ± 18.3 ^C,e^	458.0 ± 14.8 ^D,f^
PB1		278.0 ± 13.2 ^A,d^	342.7 ± 12.5 ^C,d,e^	328.0 ± 15.1 ^B,d^	412.6 ± 10.3 ^D,e^
PB2		259.2 ± 10.6 ^B,c,d^	331.2 ± 10.7 ^D,d^	213.0 ± 12.7 ^A,c^	325.3 ± 7.2 ^D,d^
PB3		237.6 ± 10.8 ^B,c^	310.6 ± 5.6 ^C,c^	178.0 ± 11.3 ^A,b^	312.7 ± 8.2 ^C,c^
PB4		175.2 ± 11.3 ^A,b^	256.6 ± 7.2 ^B,b^	166.0 ± 12.2 ^A,a,b^	265.2 ± 4.6 ^B,b^
PB5		150.8 ± 8.7 ^A,a^	223.3 ± 8.0 ^B,a^	152.0 ± 10.7 ^A,a^	212.8 ± 9.4 ^B,a^

CP—Control pasta, PB1, PB2, PB3, PB4, PB5, pasta with 1%, 2%, 3%, 4% and 5% of dried and powered banana, respectively. d.m. (dry mass). DPPH, 1,1-diphenyl-2-picrylhydrazylradical, ability to scavenge free DPPH radicals (EC50). Mean ± SD, *n* = 3, separate statistical analyses of TPC, ABTS and DPPH were performed, different small letters in the columns of the table mean significant differences between means (*p* < 0.05), different capital letters in the lines of table mean significant differences between means (*p* < 0.05).

**Table 5:** Results for cooking properties of pasta samples.

Parameter/Sample	CP	PB1	PB2	PB3	PB4	PB5
OCT (min)	6.5 ± 0.2 ^b^	6.5 ± 0.3 ^b^	5.5 ± 0.4 ^a,b^	5.5 ± 0.5 ^a,b^	4.5 ± 0.5 ^a^	4.5 ± 0.5 ^a^
WI (kg CP/kg RP)	2.88 ± 0.007 ^a^	3.08 ± 0.002 ^b^	3.42 ± 0.002 ^c^	3.49 ± 0.001 ^d^	3.97 ± 0.001 ^f^	3.55 ± 0.001 ^e^
CL (%)	5.2 ± 0.07 ^a^	5.4 ± 0.18 ^a,b^	5.5 ± 0.11 ^b,c^	5.7 ± 0.13 ^c^	6.1 ± 0.10 ^d^	6.4 ± 0.14 ^e^

OCT—Optimum cooking time (min), WI—Weight increase index, CL—Cooking loss, CP—Control pasta, RP—Raw pasta PB1, PB2, PB3, PB4, PB5, pasta with 1%, 2%, 3%, 4% and 5% of dried and powered banana, respectively. Mean ± SD, *n* = 3. Values followed by the same letter in the same row are not significantly different (*p* < 0.05).

**Table 6:** Results of dried banana sensory evaluation.

Sensory Attribute	Smell	Taste	Colour	Appearance	Texture
BP	6.70 ± 0.09	6.41 ± 0.11	6.22 ± 0.02	6.50 ± 0.13	6.38 ± 0.10

**Table 7:** Results of cooked pasta sensory evaluation.

Sample	Smell	Taste	Colour	Firmness	Adhesiveness	Overall Acceptability
CP	6.01 ± 0.10 ^d^	6.30 ± 0.10 ^d^	6.82 ± 0.03 ^d^	6.31 ± 0.12 ^bc^	6.48 ± 0.12 ^cd^	6.52 ± 0.14 ^e^
PB1	5.91 ± 0.12 ^d^	5.94 ± 0.07 ^c^	6.56 ± 0.15 ^d^	6.41 ± 0.10 ^c^	6.59 ± 0.10 ^d^	6.12 ± 0.16 ^d^
PB2	6.19 ± 0.10 ^d^	6.04 ± 0.15 ^cd^	5.68 ± 0.16 ^c^	6.15 ± 0.05 ^b^	6.33 ± 0.06 ^c^	5.93 ± 0.21 ^cd^
PB3	6.22 ± 0.07 ^d^	6.22 ± 0.16 ^cd^	5.52 ± 0.16 ^c^	6.41 ± 0.10 ^c^	6.41 ± 0.10 ^cd^	5.82 ± 0.26 ^c^
PB4	5.16 ± 0.05 ^b^	5.49 ± 0.10 ^b^	4.19 ± 0.12 ^b^	5.71 ± 0.10 ^a^	6.12 ± 0.03 ^b^	5.12 ± 0.14 ^b^
PB5	4.80 ± 0.10 ^a^	4.57 ± 0.06 ^a^	3.16 ± 0.12 ^a^	5.52 ± 0.07 ^a^	5.67 ± 0.06 ^a^	4.38 ± 0.12 ^a^

CP—Control pasta, PB1, PB2, PB3, PB4, PB5, pasta with 1%, 2%, 3%, 4% and 5% of dried and powered banana, respectively. Mean ± SD, *n* = 3, values followed by the same letter in the same columns are not significantly different (*p* < 0.05).
